# Successful Management of an Extensive Class V Carious Lesion With Dehiscence and Loss of the Attachment Apparatus: A Case Report With Six-Month Follow-Up

**DOI:** 10.7759/cureus.69047

**Published:** 2024-09-09

**Authors:** Rajat Sharma, Dax Abraham, Monika Tandan

**Affiliations:** 1 Conservative Dentistry and Endodontics, Manav Rachna Dental College, Faridabad, IND

**Keywords:** alveolar dehiscence, attachment apparatus, class v carious lesion, platelet-rich fibrin (prf), resin modified glass ionomer cement

## Abstract

The present case report presents a rare instance of a Class V carious lesion combined with alveolar dehiscence in a non-vital maxillary central incisor without gingival recession. A 25-year-old male exhibited symptoms, including a blackened tooth (#21), pus discharge, and tenderness, with clinical examination revealing a Class V carious lesion and partial dehiscence of the labial cortical plate, confirmed by radiographic and cone-beam computed tomography (CBCT) imaging. Treatment involved a two-phase approach: endodontic therapy with root canal instrumentation and obturation using a bioceramic sealer, followed by surgical intervention that included carious lesion removal, restoration with resin-modified glass ionomer cement (RMGIC), and application of a platelet-rich fibrin (PRF) membrane. PRF was selected for its regenerative properties, promoting bone healing and tissue repair, and was particularly beneficial in extensive lesions. Post-treatment follow-up at six months demonstrated complete soft tissue healing, reduced probing depth, and significant bone regeneration. This case illustrates the effective management of a complex dental condition through a combined endodontic and periodontal approach enhanced by PRF therapy, yielding favorable clinical and radiographic outcomes.

## Introduction

Class V carious lesions can only be seen in the enamel or begin on the root surfaces, frequently following gingival recession. Poor dental hygiene, xerostomia (autosomal or brought on by systemic illnesses or drugs), excessive consumption of fizzy beverages and sports drinks (with high sugar content and chemically eroded dentin), and orthodontic brackets are among the etiological factors of Class V carious lesions [1,2].

Dehiscence is defined by the loss of the marginal bone and the facial or lingual alveolar cortical plate, which causes the root surface to become denuded. This defect is commonly observed as an oval shape that extends apically from the cementoenamel junction (CEJ). Numerous factors can contribute to dehiscence defects, such as calculus deposits at the root surface under thin gingiva, developmental anomalies, root prominences, tooth malposition, tooth/jaw ratio, endo-perio lesions, calculus infections over time, periodontal disease, traumatic occlusion, and iatrogenic causes like orthodontic procedures [3,4]. The prevalence of dehiscence is highest in mandibular canines (44.26%), followed by maxillary canines (28.69%), and is lowest in the maxillary central incisors (9.84%) [5].

Our case is unique, as both a Class V carious lesion and dehiscence were present concomitantly in a non-vital maxillary central incisor without gingival recession. It’s rare that such a lesion proceeds from enamel to the root surface without gingival recession.

## Case presentation

**Figure 1:**
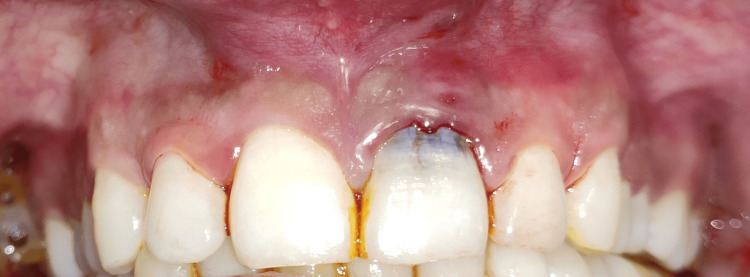
Pre-operative photograph showing Class V carious lesion, gingival redness, bleeding, and a dento-alveolar draining sinus.

A 25-year-old male patient in good health arrived with symptoms, including bleeding and pus discharge from the left upper anterior gums, and blackening of the upper left front teeth that had been present for two weeks. Upon detailed questioning, the patient reported no history of any trauma to the affected area. An intraoral clinical examination (Figure 1) showed that tooth #21, with a normal-shaped crown, had a Class V smooth carious lesion. There was no gingival recession, but gingival redness, bleeding on probing, and a dento-alveolar draining sinus were present. A 10 mm probing depth indicated clinical attachment loss. Transgingival probing for bone sounding raised the possibility that tooth #21 had a dehiscence defect. The preservation of the interproximal bone tissue between teeth #21 and #22 suggested that the lesion was contained to the facial surface of the involved tooth. Tooth #21 was tender on percussion, and electric pulp testing (EPT) showed a delayed response, indicating progression toward non-vitality.

**Figure 2:**
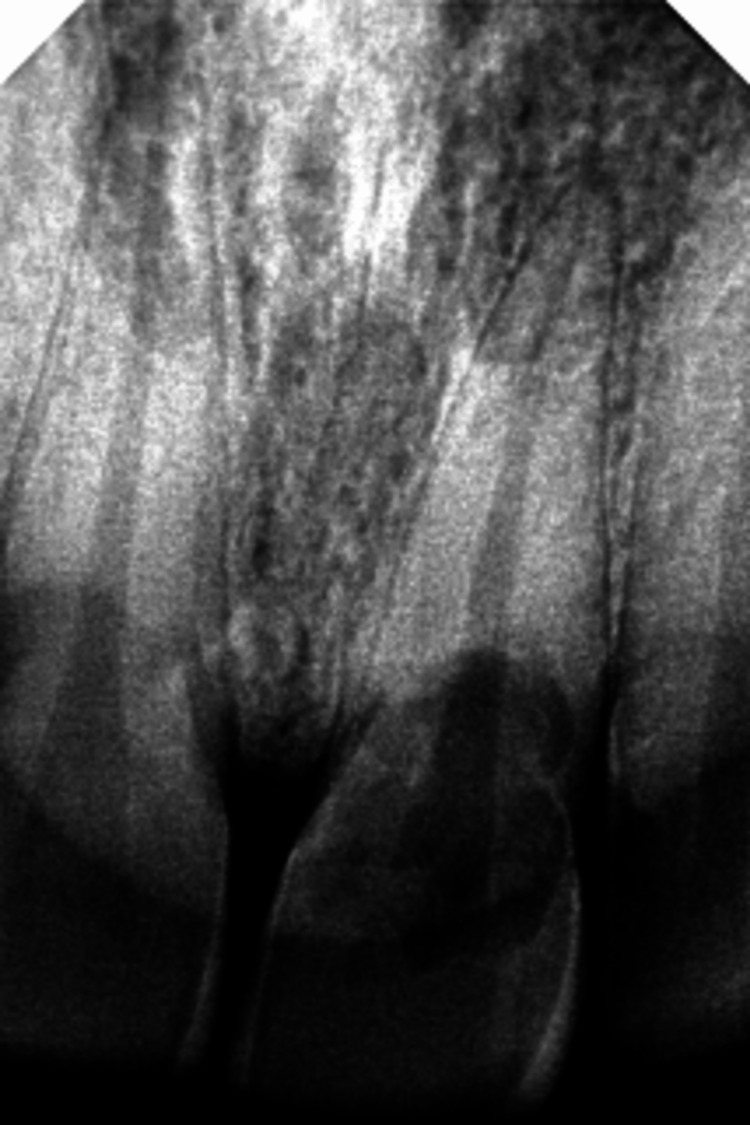
Pre-operative intra-oral periapical radiograph (IOPAR) showing periodontal ligament (PDL) widening, with loss of lamina dura and a radiolucent lesion from the middle third of the crown extending to the cervical third of the root in relation to tooth #21, overlapping the root canal without any change in the outline of the root canal.

**Figure 3:**
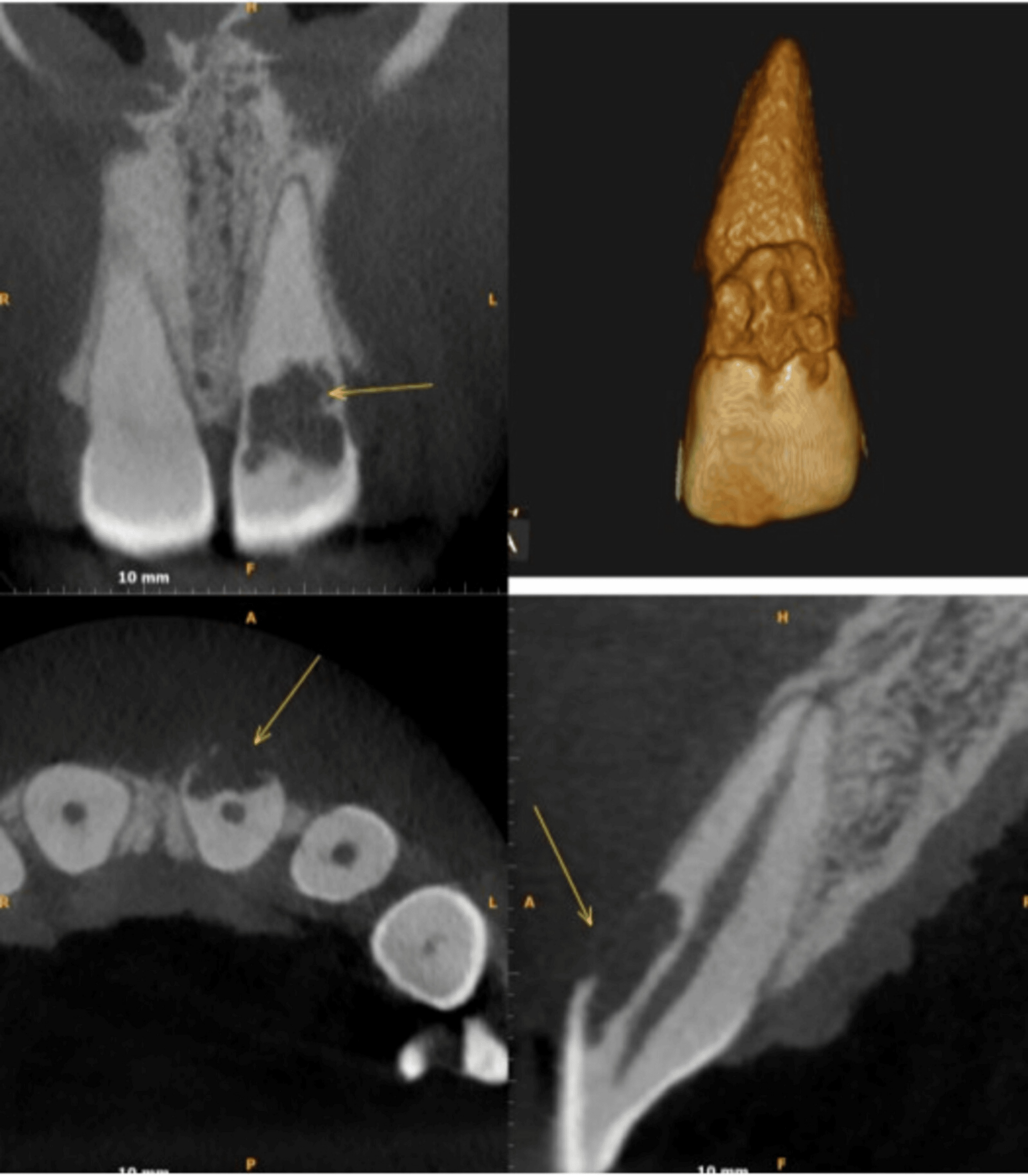
Cone beam computed tomography (CBCT) revealed an ill-defined hypodense lesion (arrows) on the labial aspect of tooth #21 at the cemento-enamel junction (CEJ) level, extending vertically to the middle one-third of the root, invading dentin, and approaching the radicular pulpal region. Partial loss of labial cortical plate indicated dehiscence in relation to tooth #21.

Pre-operative intraoral periapical radiograph (IOPAR) examination (Figure 2) revealed apical periodontal ligament (PDL) widening with loss of lamina dura and a radiolucent lesion from the middle third of the crown extending to the cervical third of the root in relation to tooth #21. This lesion overlapped the root canal, but there was no change in the root canal morphology. To ascertain the possibility of dehiscence, a cone-beam computed tomography (CBCT) scan of the maxillary anterior region was taken. CBCT (Figure 3) revealed an ill-defined hypodense lesion observed at the labial aspect of tooth #21 at the CEJ level, extending vertically to the middle one-third of the root, invading dentin and approaching the radicular pulpal region. Partial loss of the labial cortical plate indicated dehiscence in relation to tooth #21.

The diagnosis of a Type V carious lesion with alveolar dehiscence of the labial cortical plate, related to chronic endodontic infection, was finalized on the basis of clinical, radiographic, and CBCT findings. Two phases of treatment were planned: endodontic and surgical.

After fully explaining the entire procedure to the patient, written informed consent was received for both the root canal therapy and the surgical operation. The patient was prescribed mouth rinses with 0.2% chlorhexidine twice a day, in addition to oral prophylaxis.

Tooth #21 was isolated under a rubber dam on the day of the endodontic treatment, and an access cavity was created. The entire process was done at 8x magnification using a dental operating microscope. The working length was determined with the aid of an apex locator and verified radiographically. Hand K-files, in combination with 3% sodium hypochlorite (NaOCl) irrigant, were used for the chemo-mechanical preparation; the canal was then flushed one last time with 17% ethylenediaminetetraacetic acid (EDTA) solution. The root canal was further disinfected with a solution of 2% chlorhexidine. After the root canal instrumentation was finished, the patient was given a calcium hydroxide dressing and was recalled one week later. Using a bioceramic sealer (BioRoot RCS; Septodont, Saint-Maur-des-Fossés, France), obturation was performed in #21 on the following visit. Composite resin was used to restore the access cavity.

**Figure 4:**
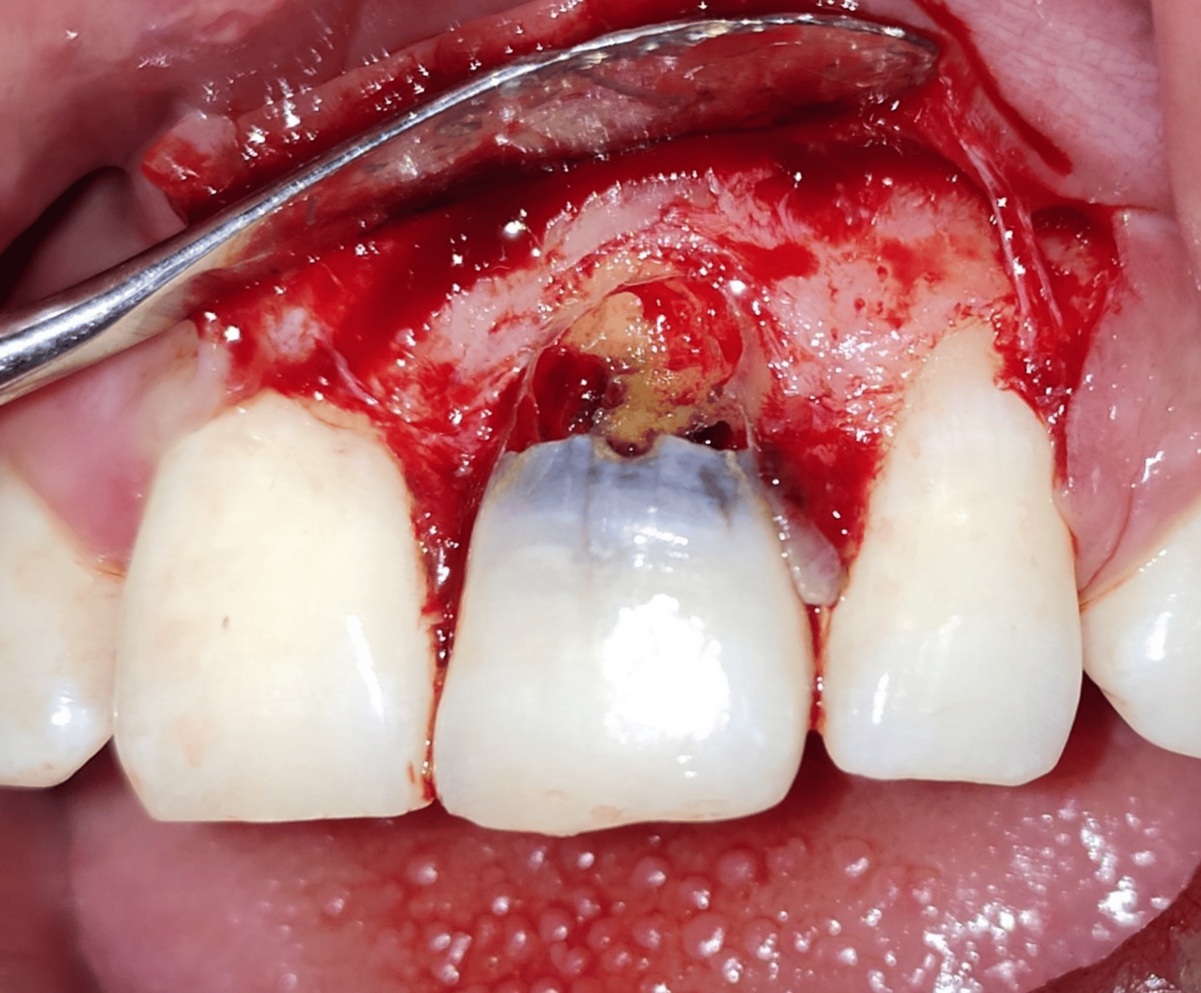
Full thickness triangular mucoperiosteal flap elevated showing dehiscence in #21, as the labial root surface was partially devoid of bone.

**Figure 5:**
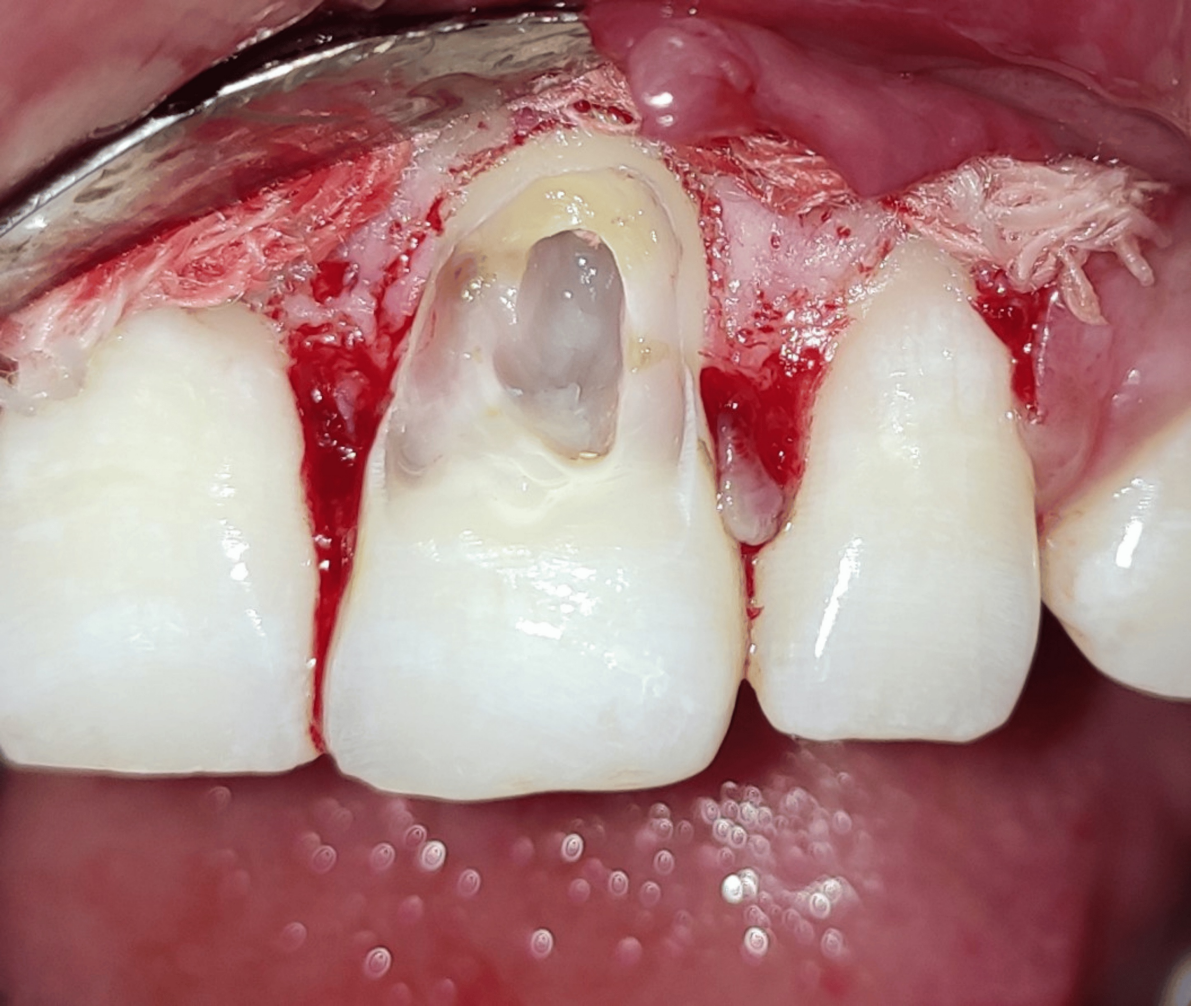
Carious lesion was removed.

**Figure 6:**
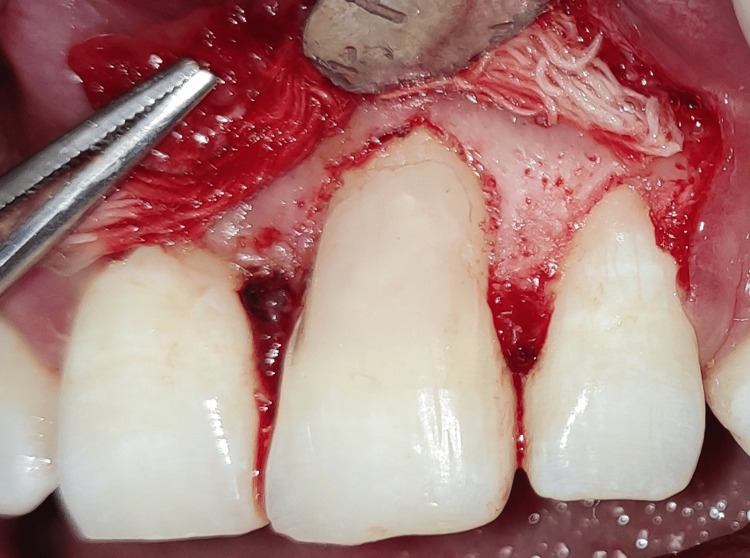
Class V lesion restored with light cure resin-modified glass ionomer cement (RMGIC).

The following day, the patient was recalled for the operation. Betadine swabs were used to disinfect the teeth, mucosal surfaces, and extraoral surfaces. Anterior superior alveolar nerve block and local infiltration were administered using a local anesthetic (lidocaine 2% and 1:80,000 epinephrine). A triangular mucoperiosteal flap was raised to maximum thickness. It was clinically proven that there was dehiscence in #21 upon reflection of the flap, as the labial root surface was entirely discolored and devoid of bone (Figure 4). The carious lesion was removed under continuous saline irrigation (Figure 5) and repaired with light-cure resin-modified glass ionomer cement (RMGIC) (GC Fuji II LC; GC Corporation, Tokyo, Japan) (Figure 6).

**Figure 7:**
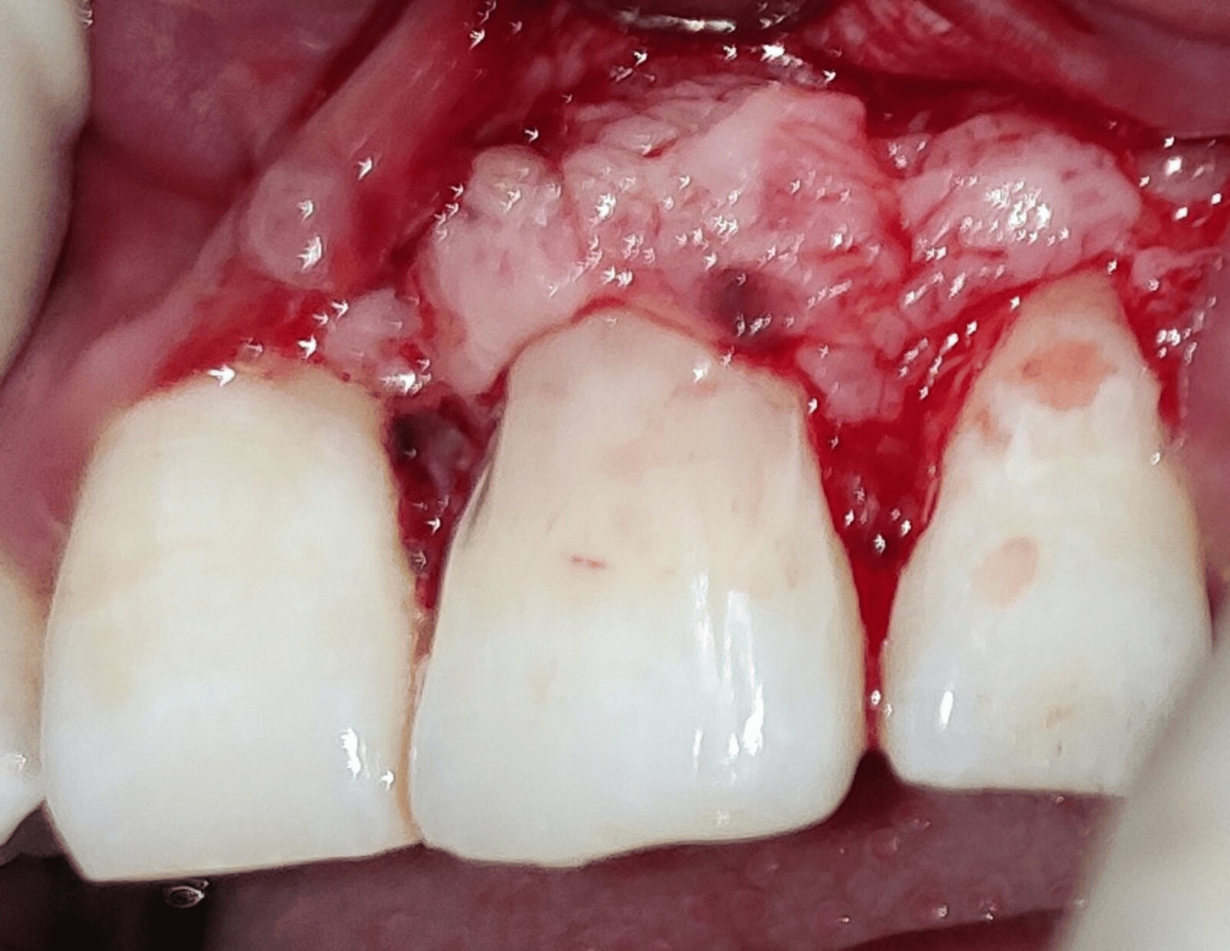
Dehiscence area was covered with a prepared platelet-rich fibrin (PRF) membrane.

**Figure 8:**
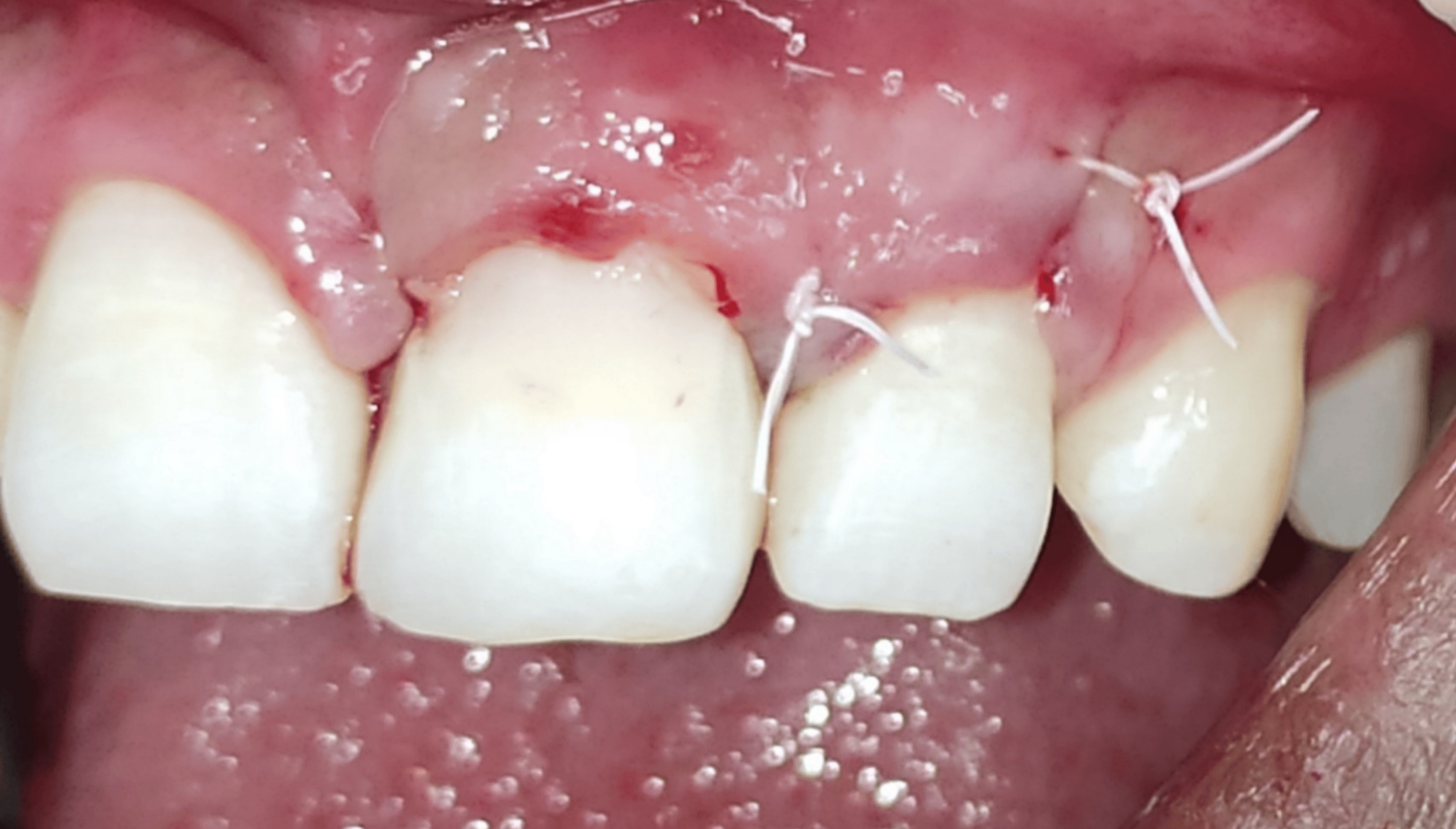
Flap repositioned and sutured with a 4-0 polytetrafluoroethylene (PTFE) suture.

**Figure 9:**
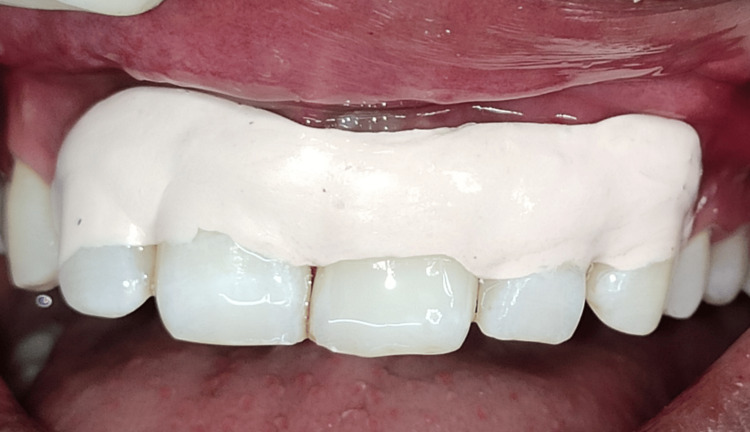
Surgical site covered with a periodontal pack (COE-PAK™).

**Figure 10:**
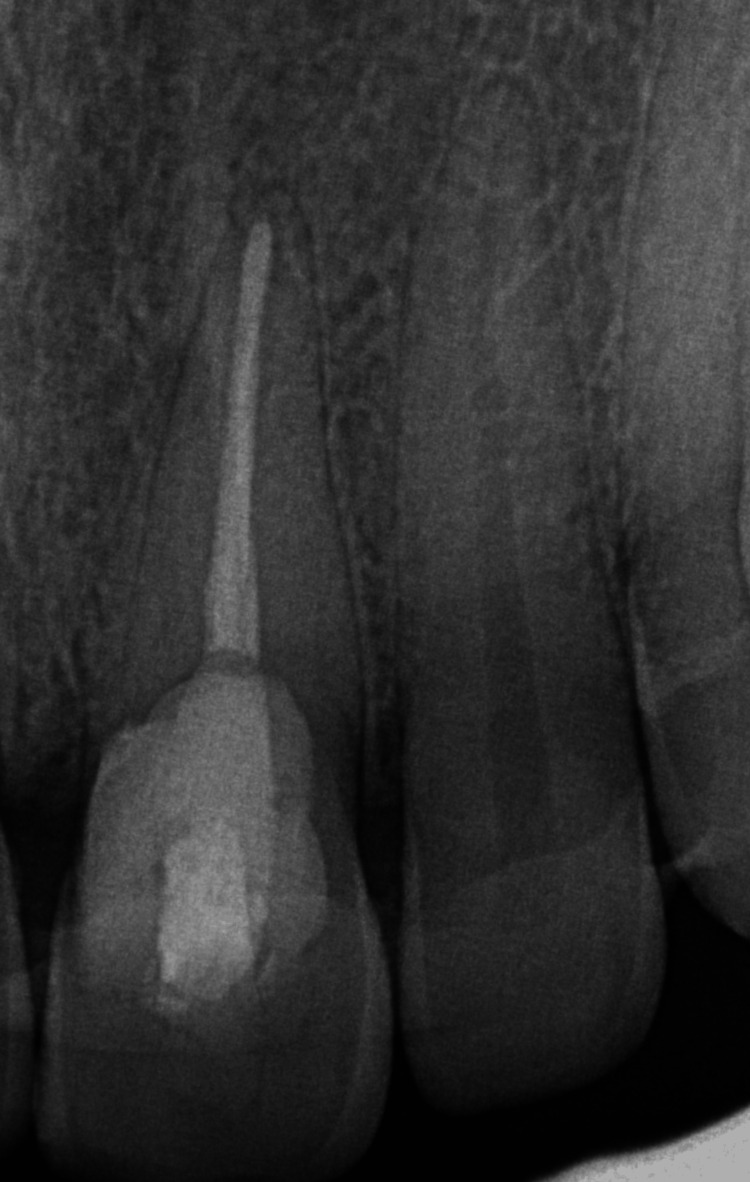
Post-operative radiograph showing root-canal obturated and restored tooth #21.

**Figure 11:**
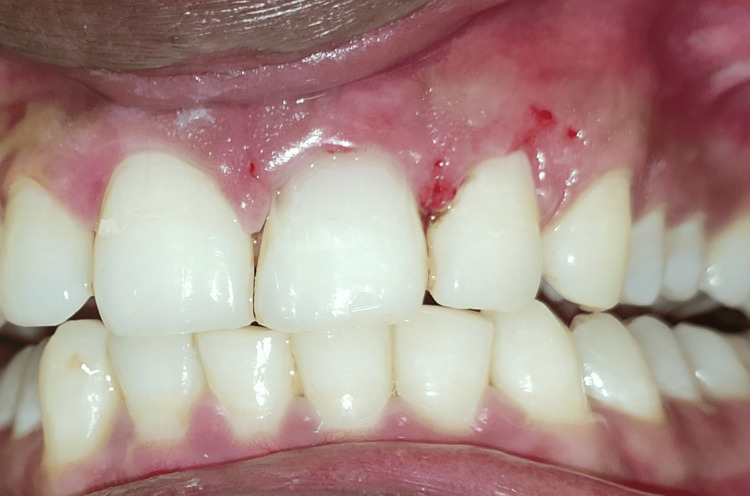
Clinical photograph showing suture removal after one week.

**Figure 12:**
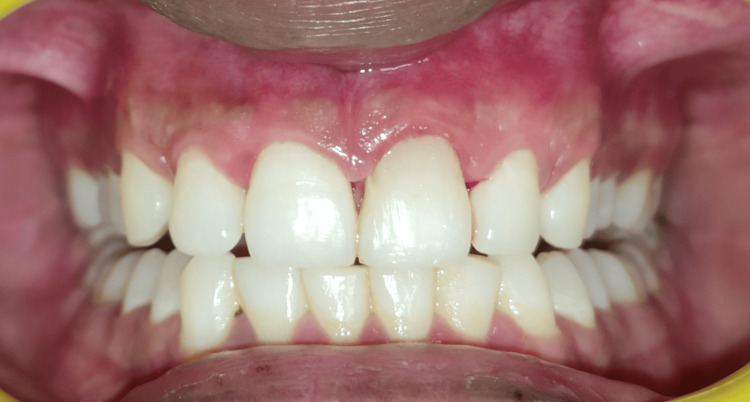
Clinical photograph at six-month follow-up revealed absence of gingival recession and complete soft tissue healing.

**Figure 13:**
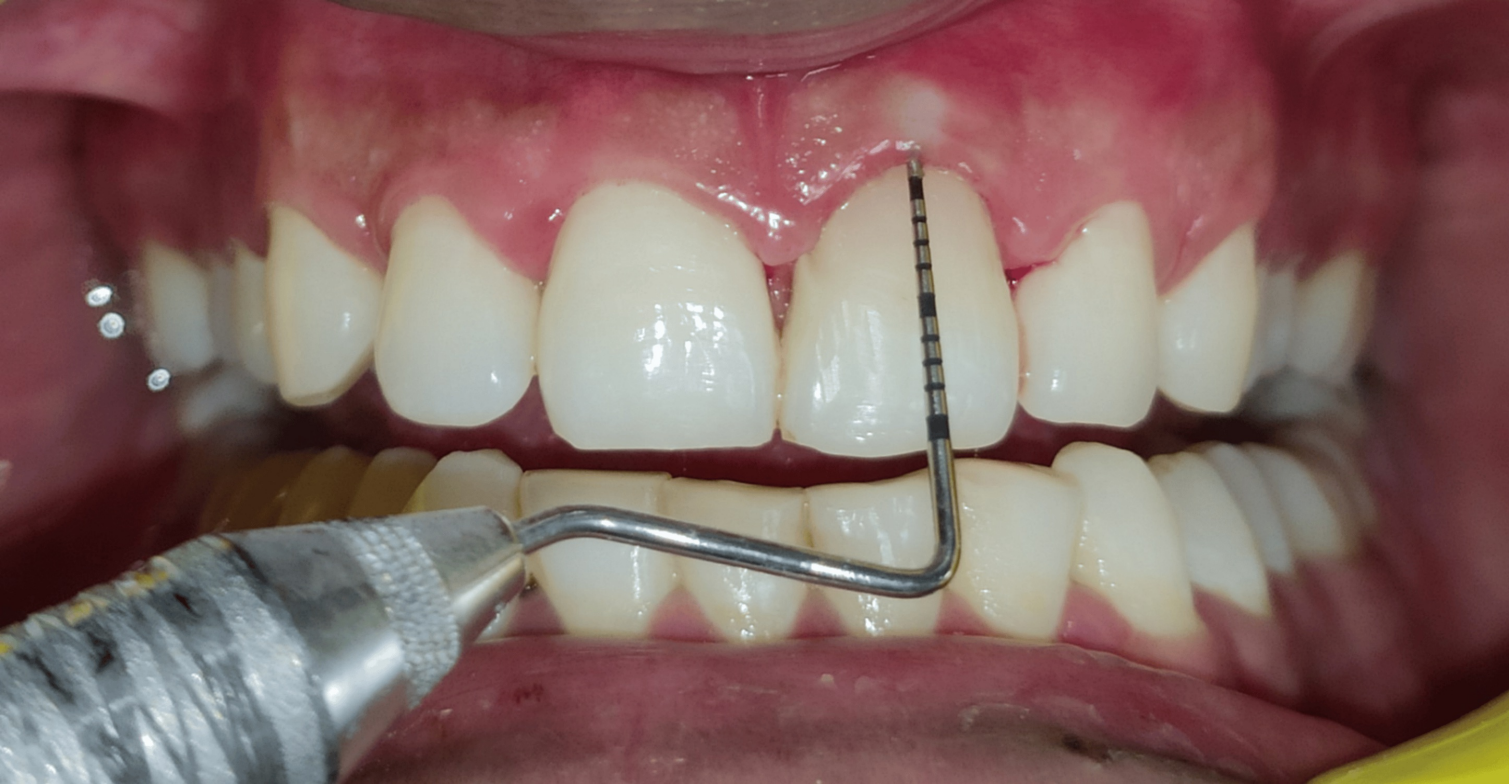
Clinical photograph after six months showing reduced probing depth of 4 mm.

**Figure 14:**
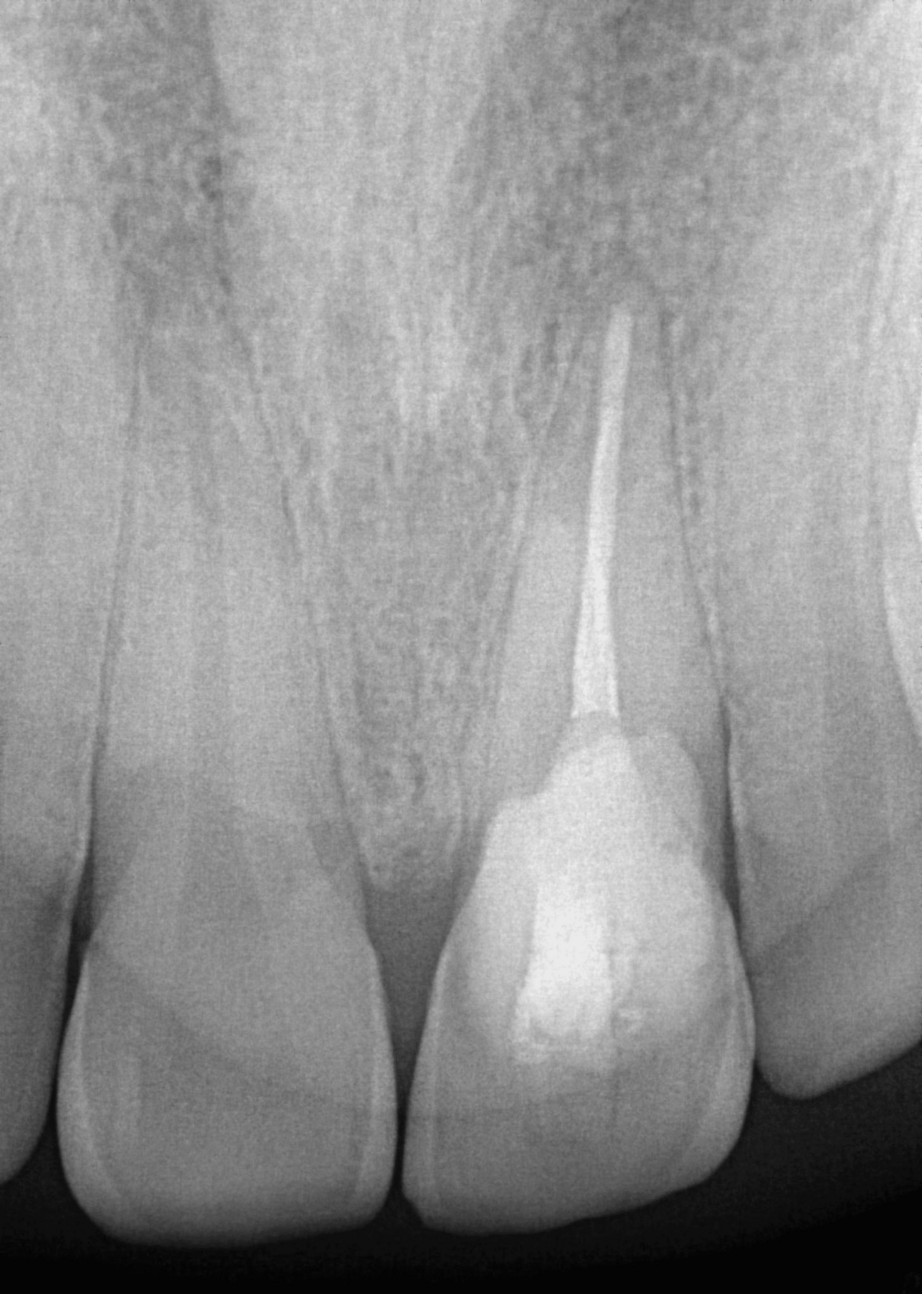
Radiograph at six-months follow-up showing significant bone repair.

The dehiscence area was overlaid with a prepared PRF membrane (Figure 7). The reflected flap was repositioned and sutured with a 4-0 polytetrafluoroethylene (PTFE) suture, using a multiple-interrupted approach (Figure 8). The surgical site was protected with a periodontal pack (COE-PAK™; GC Corporation) (Figure 9). A postoperative radiograph was taken (Figure 10). Antibiotics, analgesics, and 0.2% chlorhexidine mouthwash were prescribed, along with postoperative instructions to the patient. After a week, the sutures were removed (Figure 11). Follow-up appointments were set for one, three, and six months later. Clinical examination at the six-month follow-up revealed the absence of gingival recession and complete soft tissue healing, with a reduced probing depth of 4 mm (Figures 12-13). Substantial bone reconstruction was observed on radiographic assessment (Figure 14).

## Discussion

Gaining adequate access to deep cervical caries can be challenging. Remaining caries, inadequate restoration adaptability, and flawed margins could arise from the inability to observe, segregate, and reach the complete lesion. Minor periodontal operations might improve visibility and access to the problematic areas. These procedures range from traditional flap surgery to gingivoplasty and crown lengthening techniques involving a single tooth. When done correctly, minor periodontal surgery, combined with restorative or endodontic treatment, can deliver outstanding and long-lasting results in troublesome areas [6].

The material options are resin-based composites or conventional/modified glass ionomer cement. Frequently, the restoration site is easily contaminated; hence, RMGIC is an ideal material. It provides a highly polished, easily cleanable restoration surface that may lessen the chance of secondary caries. Glass ionomer cements treated with resins are biocompatible and exhibit chemical adhesion to the tooth structure. They are suited to subgingival and furcation restorations because of their decreased curing shrinkage and insolubility in oral fluids. However, RMGIC may have limitations in terms of mechanical strength and wear resistance compared to alternatives like Biodentine for root restoration, which offers superior bioactivity and dentin-like mechanical properties, and composite resin for crown restorations, which provides better durability and esthetics but is more technique-sensitive. The histomorphometric reaction of periodontal tissues to various restorative materials investigated in animals, such as composite and RMGIC, shows no difference in the inflammatory tissue response between restorations and control areas. The lack of an inflammatory response to the biocompatible materials was attributed to meticulous finishing and polishing prior to flap closure and control of plaque during the healing phase, both of which are crucial for the successful resolution of clinical cases [7]. 

Carious Class V cervical lesions are commonly attributed to chronic plaque accumulation at the cervical third of the tooth, coupled with a high intake of fermentable carbohydrates. An additional risk factor is a reduction in salivary flow and quality due to xerostomia, which compromises the mouth’s ability to buffer acids and maintain oral health [8].

Dehiscence, an alveolar bone defect, is indicated by the absence of a minimum of 4 mm of cortical bone apical to the margin of the interproximal bone [9]. Many studies have reported geographical variations in anomalies of the alveolar bone, with an overall frequency of dehiscence found in skulls ranging from 0.99% to 53.62% [10]. These alveolar defects arise from different predisposing factors, such as the decreased thickness of the alveolar bone, labial placement of the tooth in the dental arch, contour of the roots, abnormal occlusal factors, orthodontic tooth movement, or periodontal and endodontic pathologies. More often than not, these conditions are commonly associated with the anterior region of the dental arch [11]. Both the patient and the dentist frequently miss these problems, especially if the patient is asymptomatic [12]. Transgingival probing aids in the dehiscence diagnosis. On traditional two-dimensional dental radiographs, dehiscence cannot be measured. In this instance, CBCT imaging was used to get around this restriction. CBCT results that showed abnormalities in both cancellous and cortical bone supported clinical findings. As a result, CBCT serves as an accurate guide for deciding on the course of treatment and the lesion's prognosis.

Root carious pulpal exposures with dehiscence are best treated through endodontic therapy combined with an open flap technique. Autologous platelet concentrates, such as platelet-rich plasma (PRP), platelet-rich fibrin (PRF), or resorbable guided tissue regeneration (GTR) membranes (synthetic or natural), can be utilized to regenerate lost periodontium. However, PRF offers several benefits, such as promoting the healing of both soft and hard tissues through its fibrin bandage and the release of growth factors like platelet-derived growth factor (PDGF) and transforming growth factor. The fibrin network in PRF supports enhanced cell growth, migration, and proliferation [13]. Despite the fact that leukocyte and platelet cytokines are crucial for PRF’s repairing ability, it has often been suggested that the fibrin matrix surrounding these components is what gives PRF its therapeutic potential [14]. PRF has the ability to recruit circulating stem cells, modulate the immune system, promote undisturbed wound closure/healing by epithelial tissues, and promote angiogenesis - all of which are critical to tissue regeneration [14]. Therefore, the three-dimensional structure of the fibrin matrix, which includes multiple growth factors and cytokines like PDGF, transforming growth factor beta-1 (TGF-β1), insulin-like growth factor (IGF), and vascular endothelial growth factor (VEGF) concurrently incorporated in the matrix, may account for the angiogenic qualities of PRF. Additionally, the fibrin matrix promotes the expression of integrin αvβ3, allowing cells to bind to fibrin, fibronectin, and vitronectin [15]. This series of events is crucial for starting the angiogenesis process and, consequently, tissue wound healing [15]. Moreover, the byproducts of fibrin degradation directly promote neutrophil migration and facilitate transmigration into the vascular endothelium, which activates neutrophils and causes them to secrete proteases that help break down the fibrin thrombus and penetrate the blood vessel basement membrane. Neutrophils become entangled in the fibrin thrombus and, via phagocytosis, the generation of harmful free radicals and digestive enzymes aid in the removal of invading microbes and pathogens from the surgical site [16]. In osteogenesis, which promotes healing and repair, macrophages present in PRF play a crucial role in the shift from inflammation to wound repair [15,16]. The reparative potential of PRF promotes the regeneration of soft tissues and ligaments, which is another intriguing finding. Increased clinical attachment levels and a much larger decrease in periodontal probing depth were observed when PRF was primarily used for intra-bony defect correction [17]. Consequently, these results show that PRF can facilitate PDL healing just as well, if not more so than widely used biomaterials. The therapeutic strategy employed in the care of this complex case has produced suitable radiographic and clinical results that align with previous research published in the literature [12,18,19]. A limitation of this case is the lack of long-term follow-up, which would be necessary to fully assess the success and stability of the treatment outcomes. Future studies with extended follow-up periods are recommended to better understand the longevity and efficacy of the applied treatment.

## Conclusions

This case report describes a unique instance in which a Class V carious lesion, dehiscence, and persistent periapical inflammation were observed in the maxillary left central incisor. It was successfully managed with a blend of periodontal and endodontic techniques. The use of RMGIC and PRF resulted in positive radiographic and clinical outcomes.
